# Epidemiological linkage between migraine and diabetes mellitus: a systematic review and meta-analysis

**DOI:** 10.1186/s10194-024-01868-2

**Published:** 2024-09-27

**Authors:** Woo-Seok Ha, Vinh Khang Nguyen, Min Kyung Chu

**Affiliations:** 1 Department of Neurology, Yonsei University College of Medicine, Seoul, Republic of Korea; 2 Department of Neurology, University Medical Center HCMC, Ho Chi Minh City, Vietnam; 3 Department of Neurology, Severance Hospital Yonsei University College of Medicine, 50-1 Yonsei-ro, Seodaemun-gu, Seoul, 03722 Republic of Korea

**Keywords:** Migraine disorders, Diabetes Mellitus, Headache, Epidemiology, Meta-analysis

## Abstract

**Background:**

This study aimed to elucidate the nature and extent of the associations between diabetes mellitus (DM) and migraine through a systematic review and meta-analysis.

**Methods:**

We searched the PubMed, Web of Science, and Scopus databases without a specified start date until June 2, 2024. Cross-sectional and cohort studies analyzing the risk of migraine in individuals with DM and vice versa were included. Studies without at least age and sex adjustments were excluded. Data were extracted to calculate odds ratios (ORs) and hazard ratios (HRs). Risk of bias was assessed using the Newcastle–Ottawa Quality Assessment Scale.

**Results:**

Eight cross-sectional studies (131,361 patients with DM and 1,005,604 patients with migraine) and four cohort studies (103,205 patients with DM patients and 32,197 patients with migraine) were included. Meta-analyses of the cross-sectional studies showed no significant overall association between DM and migraine. Subgroup analyses revealed that type 1 diabetes reduced the odds of having migraine (OR 0.48, 95% confidence interval [CI] 0.30–0.77), while migraine without aura (MO) increased the odds of having DM (OR 1.19, 95% CI 1.02–1.39). The cohort studies indicated that DM decreased the risk of developing migraine (HR 0.83, 95% CI 0.76–0.90), and a history of migraine increased the risk of developing DM (HR 1.09, 95% CI 1.01–1.17).

**Conclusions:**

DM, particularly type 1 diabetes, is negatively associated with migraine occurrence, whereas migraine, especially MO, is positively associated with DM occurrence. However, most of the results remained at a low or very low level of evidence, indicating the need for further research.

**Supplementary Information:**

The online version contains supplementary material available at 10.1186/s10194-024-01868-2.

## Background

Migraine and diabetes mellitus (DM) are two prevalent chronic conditions that significantly impact individuals’ quality of life and pose considerable public health burdens. Migraine, characterized by recurrent headaches and often accompanied by nausea, photophobia, and phonophobia, affects approximately 12% of the global population [1]. DM, a metabolic disorder marked by chronic hyperglycemia due to insulin resistance or deficiency, affects over 400 million people worldwide [2]. Both conditions are associated with various comorbidities and have complex pathophysiological mechanisms [3].

Epidemiological studies have suggested potential bidirectional associations between migraine and DM [4]. Understanding these associations is crucial for developing comprehensive management strategies for patients with each or both conditions [5]. Despite the growing interest in the interplay between migraine and DM, existing research has reported conflicting results [4]. Differences in study design, population characteristics, diagnostic criteria, and statistical adjustments have contributed to these inconsistencies. Therefore, a systematic review and meta-analysis of the existing epidemiological studies are essential to clarify the nature and extent of the association between migraine and DM.

In this study, we aimed to synthesize the current evidence on the bidirectional relationship between migraine and DM by analyzing data from both cross-sectional and cohort studies. Examining the associations between these conditions will provide insights that could inform clinical practice and guide future research into the mechanisms underlying their interaction.

## Methods

This systematic review and meta-analysis followed the Preferred Reporting Items for Systematic Reviews and Meta-Analyses guidelines [6]. The study was registered with PROSPERO (registration number CRD42024536196).

### Search strategies and selection criteria

**Fig. 1:**
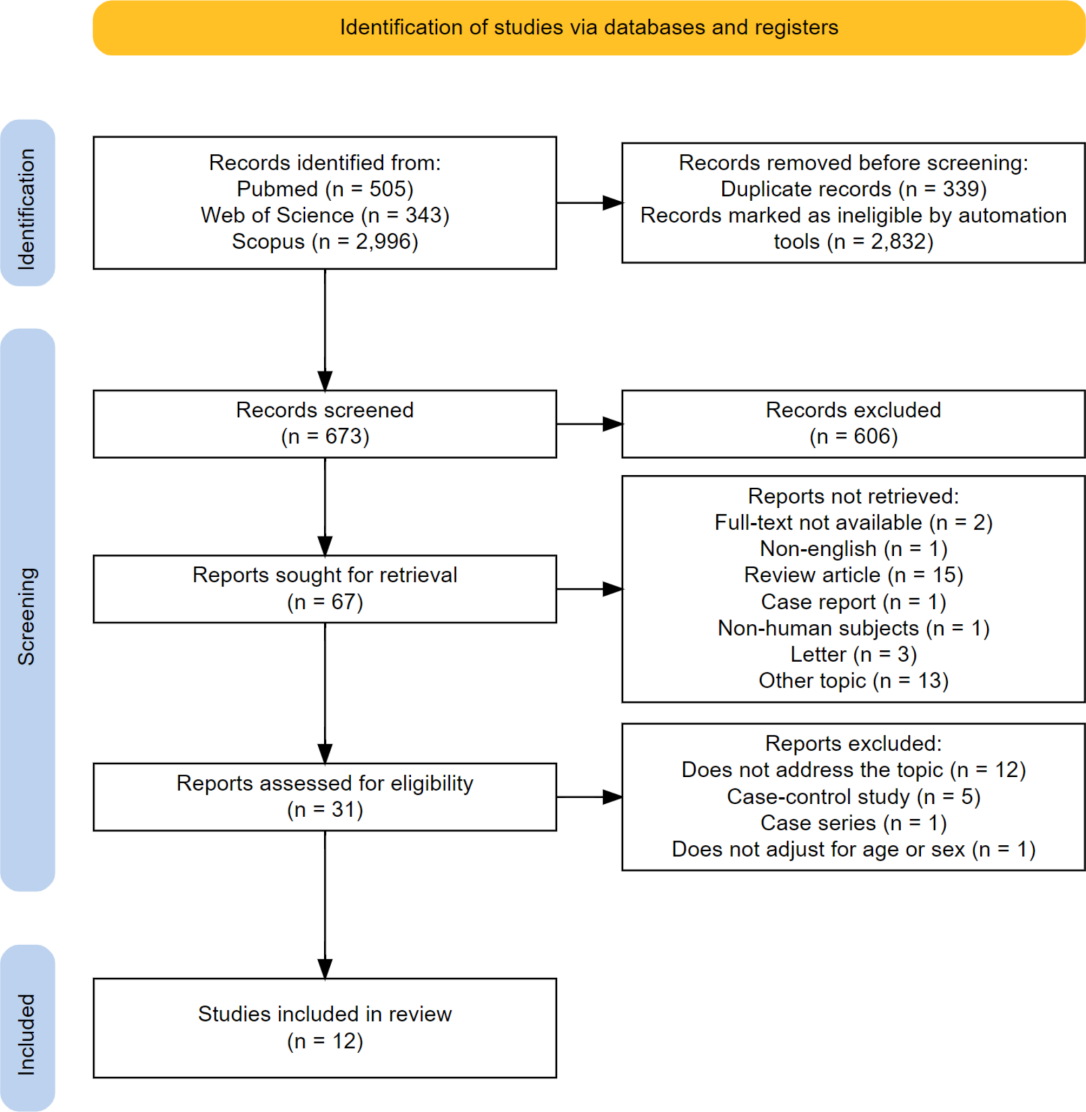
PRISMA literature search flowchart

Two reviewers (WSH and VKN) independently searched the PubMed, Web of Science, and Scopus databases, with no specified start date, until June 2, 2024. We used the search terms “diabetes mellitus” and “migraine”, combining medical subject headings (MeSH) and non-MeSH terms with Boolean operators (OR and/or AND). Supplementary Material S1 provides full details of the search strategy. Duplicate articles and articles that did not include the terms migraine or diabetes in their abstracts were removed before screening automatically. The initial search yielded 673 records. We excluded records that were not relevant to the topic based on their article titles and selected 67 records to further review their abstracts. We excluded review articles, letters, case reports, non-English articles, and articles with non-relevant topics. The full texts of the remaining 31 articles were then screened against the inclusion criteria. We included cross-sectional or cohort studies that analyzed either the risk of migraine in individuals with DM or the risk of DM in individuals with migraine, each compared to a control group without the respective conditions. We excluded case-control studies and studies that did not perform at least age and sex adjustment or stratification, given the characteristics of the two conditions. The list of studies that were excluded during the full-text screening is provided in Supplementary Material S2. Finally, 12 studies on the epidemiological linkage between migraine and DM were included (Fig. 1).

### Data extraction and analysis

Data were manually extracted from the included studies into a Microsoft Excel spreadsheet. We extracted odds ratios (ORs) with 95% confidence intervals (CIs) for cross-sectional studies, as well as hazard ratios (HRs) with 95% CIs for cohort studies. When a study provided outcomes adjusted for other covariates along with age and sex, we included those adjusted outcomes. If a study did not present pooled ORs or HRs but only provided outcomes for subgroups (e.g., type 1 and type 2 diabetes, or migraine with aura [MA] and without aura [MO]), we calculated the pooled ORs and HRs using a meta-analytic approach through inverse-variance weighting. Similarly, if a study did not adjust for age and sex but provided age- or sex-stratified outcomes, we used the same approach to calculate the pooled ORs for each stratum, then combined them to derive age- and sex-adjusted outcome results.

The meta-analyses were conducted as four separate analyses based on whether the studies were cross-sectional or cohort studies and whether they examined the risk of migraine in DM or the risk of DM in migraine. Meta-analyses were performed using RevMan Web 7.7.2 (Cochrane Collaboration, London, United Kingdom). If a study reported outcomes for subgroups, these results were included in the subgroup analysis. The I^2^ measure of heterogeneity was calculated for each meta-analysis, and funnel plots were generated to assess publication bias for the meta-analyses that included at least five studies.

### Risk of bias and quality of evidence

The risks of bias for the included studies were independently assessed by two authors (WSH and VKN) using the Newcastle–Ottawa Quality Assessment Scale for the cohort studies and an adapted form for the cross-sectional studies (Supplementary Material S3) [7]. Discrepancies were resolved by discussions with a third reviewer (MKC). We used the Grading of Recommendations Assessment, Development and Evaluation (GRADE) system to rate the quality of the body of evidence presented in this study [8].

## Results

### Cross-sectional studies

Eight cross-sectional studies were included in this study, of which four studies evaluated the odds of having migraine in individuals with DM, and four studies evaluated the odds of having DM in individuals with migraine (Table 1). Detailed risk of bias assessment scores is provided in Supplementary Material S4. Heterogeneity was observed among the methods of the included studies in terms of study populations and adjusted variables. Most studies used self-reported DM diagnosis, while migraine diagnosis was based on surveys using the International Classification of Headache Disorders criteria. A study by Minen et al. combined individuals with migraine and severe headaches [9]. Hagen et al. derived two sets of results from two databases, the Nord-Trøndelag Health Study (HUNT study) 2 and the HUNT3 study. One set of results was obtained from participants in both the HUNT2 and HUNT3 studies, while the other set was obtained from participants in only the HUNT3 study. As data from the HUNT2 study were also used in another study by Aamodt et al., we only utilized the results obtained from the HUNT3 study reported by Hagen et al. [10, 11].

**Table 1:** Cross-sectional studies investigating the association between migraine and diabetes mellitus

Association	Author, year	Country	Participants of database	Patients, *n*	Controls, *n*	Diagnosis of DM	Diagnosis of migraine	Adjustments	NOS
Odds of having migraine in DM	Aamodt et al., 2007 [10]	Norway	Aged ≥ 20	DM, *n* = 1,499 Type 1 diabetes, *n* = 179 Type 2 diabetes, *n* = 870	49,750	Self-report	ICHD-1	Age, sex, and education	6
	Berge et al., 2013 [12]	Norway	All Norwegians	DM, *n* = 124,649	4,515,570	Use of antidiabetic drugs	Use of ergotamine and/or triptans	Age and sex	7
	Hagen et al., 2018 [11]	Norway	Aged ≥ 20	DM, *n* = 1,772 Type 1 diabetes, *n* = 131 Type 2 diabetes, *n* = 1,436 LADA, *n* = 81 Unclassified DM, *n* = 124	39,584	Self-report	ICHD-2	Age, sex, education, and smoking	6
	López-de-Andrés et al., 2018 [13]	Spain	Aged ≥ 40	DM, *n* = 3,441	3,441	Self-report	Self-report	Age, sex, respiratory disease, mental disorder, neck pain, low back pain, and use of pain drugs	5
Odds of having DM in migraine	Bigal et al., 2010 [14]	USA	Aged ≥ 18	Migraine, *n* = 6,102 MA, *n* = 2,000 MO, *n* = 4,102	5,243	Self-report	ICHD-2	Age and sex	6
	Minen et al., 2019 [9]	USA	Aged ≥ 18	Migraine or severe headache, *n* = 15,853	88,990	Self-report	Self-report	Age, sex, ethnicity, and survey year	5
	Patel et al., 2019 [15]	USA	Hospitalized, aged ≥ 18	Migraine, *n* = 983,065	55,516,723	ICD-9-CM code	ICD-9-CM code	Age, sex, ethnicity, hospitalization variables, hospital-level variables, and CCI	6
	Schramm et al., 2021 [16]	Germany	Aged 45 to 75	Migraine, *n* = 584 MA, *n* = 168 MO, *n* = 416	634	Either of 1) self-report, 2) used antidiabetic drugs, or 3) fasting serum glucose ≥ 200 mg/dL	ICHD-2	Age and sex	6

DM, diabetes mellitus; NOS, Newcastle–Ottawa Scale; ICHD, the International Classification of Headache Disorders criteria; LADA, latent autoimmune diabetes in adults; MA, migraine with aura; MO, migraine without aura; ICD-9-CM, International Classification of Diseases, 9th Revision, Clinical Modification; CCI, Charlson’s comorbidity index.

### Odds of having migraine in individuals with DM

**Fig. 2:**
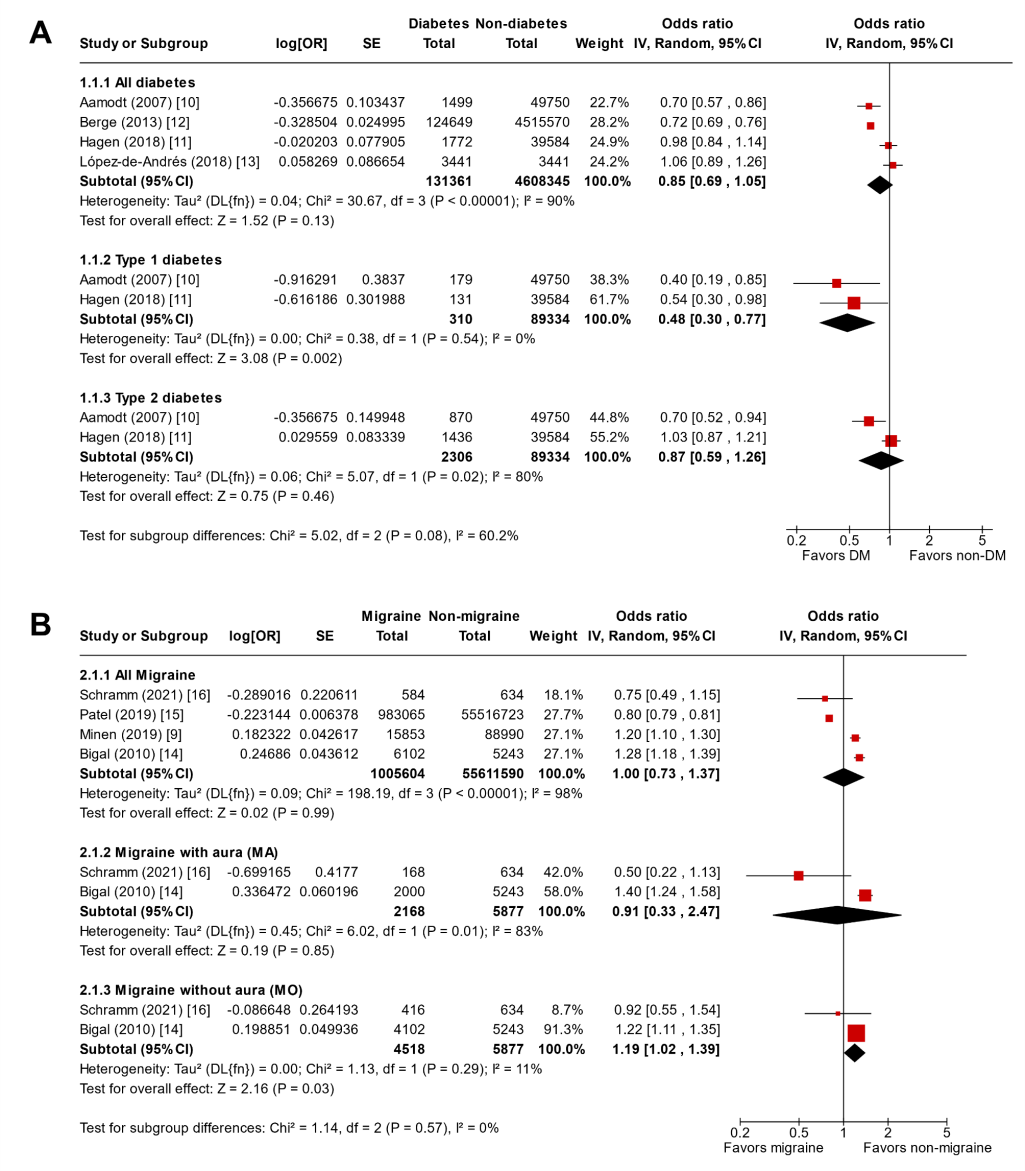
Forest plot for the meta-analysis of cross-sectional studies showing the (**A**) odds of having migraine in individuals with DM and (**B**) odds of having DM in individuals with migraine OR, odds ratio; SE, standard error; DM, diabetes mellitus; IV, inverse-variance; CI, confidence interval

In the meta-analysis of four studies, involving 131,361 individuals with DM and 4,608,345 individuals without DM, DM did not significantly affect the odds of having migraine (OR 0.85, 95% CI 0.69–1.05; Fig. 2A). Substantial heterogeneity was observed among the studies (I^2^ = 90%). Aamodt et al. and Hagen et al. analyzed individuals with type 1 and type 2 diabetes separately, and the subgroup analysis reflecting these two studies showed that type 1 diabetes was associated with significantly lower odds of having migraine (OR 0.48, 95% CI 0.30–0.77), while type 2 diabetes showed no significant association with the odds of having migraine (OR 0.87, 95% CI 0.59–1.26) [10, 11]. Supplementary Material S5 presents the certainty of evidence based on the GRADE framework. The odds of having migraine in patients with DM were considered as very low-quality evidence, except in those with type 1 diabetes, where the evidence was considered as low-quality. The funnel plot of these four studies is illustrated in Supplementary Material S6.

### Odds of having DM in individuals with migraine

In the meta-analysis of four studies, involving 1,005,604 individuals with migraine and 55,611,590 individuals without migraine, migraine did not significantly affect the odds of having DM (OR 1.00, 95% CI 0.73–1.37; Fig. 2B). However, substantial heterogeneity was observed among studies (I^2^ = 98%). Bigal et al. and Schramm et al. analyzed individuals with MA and MO separately, and the meta-analysis reflecting these two studies showed that MO was associated with increased odds of having DM (OR 1.19, 95% CI 1.02–1.39), while MA showed no significant association with having DM (OR 0.91, 95% CI 0.33–2.47) [14, 16]. The odds of having DM in patients with migraine were assessed as being based on very low-quality evidence. The funnel plot for the four studies is illustrated in Supplementary Material S7.

### Cohort studies

Four cohort studies were included (Table 2). Among the two studies that evaluated the risks of developing migraines in individuals with DM, Antonazzo et al. provided separate assessments for individuals with type 1 and type 2 diabetes, whereas Wu et al. followed up on only individuals with type 2 diabetes [17, 18]. Both Fagherazzi et al. and Burch et al. evaluated the risks of developing DM in individuals with migraine [19, 20]. Both recruited female-only cohorts, and they analyzed individuals with active migraine and those with a history of migraine separately at baseline.

**Table 2:** Cohort studies investigating the association between migraine and diabetes mellitus

Association	Author, year	Country	Participants of cohort	Follow up period	Patients, *n*	Controls, *n*	Diagnosis of DM	Diagnosis of migraine	Adjustments	NOS
Risks of developing migraine in DM	Antonazzo et al., 2018 [17]	Norway	Aged < 80	10 years	Type 1 diabetes, *n* = 7,883 Type 2 diabetes, *n* = 93,600	4,184,718	Use of antidiabetic drugs	Use of ergotamine and/or triptans	Age, sex, and education	8
	Wu et al., 2024 [18]	China	Aged ≥ 45	4 years	Type 2 diabetes, *n* = 1,722	8,151	Either of 1) fasting blood glucose levels ≥ 126 mg/dl or 2) HbA1c ≥ 6.5%	ID Migraine Questionnaire	Age, sex, education, BMI, residence, marital status, drinking, smoking, socializing, exercising, hypertension, depression, and sleep duration	6
Risks of developing DM in migraine	Burch et al., 2012 [19]	USA	Women, aged ≥ 45	Mean of 14.6 years	Active MA, *n* = 2,014 Active MO, *n* = 3,048 History of migraine, *n* = 2,087	31,471	Both 1) self-report and 2) physician-administered telephone interviews using the American Diabetes Association diagnostic criteria or a self-administered supplemental questionnaire	Self-report, with further ICHD-1 based survey	Age, (sex)	6
	Fagherazzi et al., 2019 [20]	France	Women, born between 1925 and 1950	10 years	Active migraine (*n* = 7,839) Prior migraine (*n* = 17,209)	49,199	Pharmacologically treated with Type 2 diabetes-specific medications	Self-report	Age, (sex)	7

DM, diabetes mellitus; NOS, Newcastle–Ottawa Scale; BMI, body mass index; MA, migraine with aura; MO, migraine without aura; ICHD, the International Classification of Headache Disorders criteria.

### Risks of developing migraine in individuals with DM

**Fig. 3:**
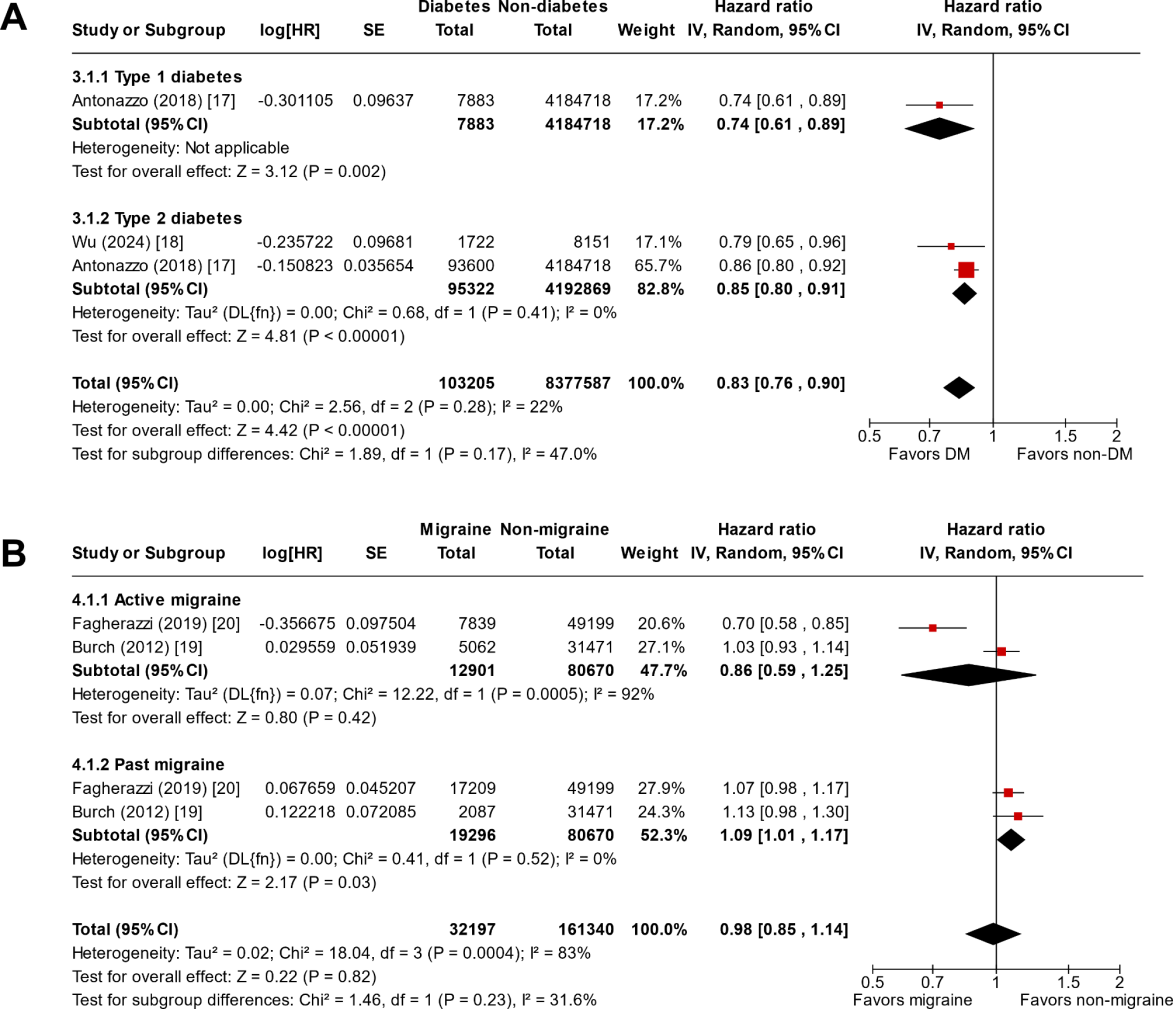
Forest plot for the meta-analysis of cohort studies showing the (**A**) risks of developing migraine in individuals with DM and (**B**) risks of developing DM in individuals with migraine HR, hazard ratio; SE, standard error; DM, diabetes mellitus; IV, inverse-variance, CI, confidence interval

The meta-analysis revealed that individuals with DM were less likely to develop migraine (HR 0.83, 95% CI 0.76–0.90; Fig. 3A). In the subgroup analysis based on the types of DM, only one study that analyzed patients with type 1 diabetes showed an HR of 0.74 (95% CI 0.61–0.89), and two studies that analyzed patients with type 2 diabetes showed an HR of 0.85 (95% CI 0.80–0.91). Low heterogeneity was observed between the two studies (I^2^ = 0%). The risks of developing migraine in patients with DM were considered as low-quality evidence.

### Risks of developing DM in individuals with migraine

The meta-analysis revealed that migraine did not have a significant impact on the development of DM (HR 0.98, 95% CI 0.85–1.14; Fig. 3B). However, when the analysis was performed based on migraine activity at baseline, individuals with a history of migraine showed a significant increase in the risk of developing DM (HR 1.09, 95% CI 1.01–1.17). While significant heterogeneity was observed between the two studies with results regarding active migraine (I^2^ = 92%), low heterogeneity was observed for the results regarding a history of migraine (I^2^ = 0%). The risks of developing DM in patients with migraine were considered as very low-quality evidence.

## Discussion

This meta-analytic review, which included both cross-sectional and cohort studies, revealed nuanced associations between DM and migraine as follows: (1) In the cross-sectional studies, no significant overall association was observed between the two conditions. However, in the subgroup analysis, patients with type 1 diabetes had reduced odds of having migraine, while patients with MO had increased odds of having DM and (2) in the cohort studies, DM, regardless of whether it was type 1 or type 2 diabetes, decreased the risks of developing migraine. Conversely, a history of migraine increased the risks of developing DM.

While the two conditions are likely interconnected through various mechanisms, our study found that DM, particularly type 1 diabetes, is negatively associated with the occurrence of migraine. Several hypotheses can be proposed as the mechanisms for this effect. First, diabetic peripheral neuropathy (DPN) may show a preventive effect on migraine. DPN mainly involves neurodegeneration of small unmyelinated or thinly myelinated fibers such as C and Aδ fibers, caused by inflammatory damage due to a hyperglycemic state and reduced oxygen delivery in patients with diabetes [21, 22]. As these nerve fibers play a crucial role in the pathogenesis of migraine, DPN may prevent migraine by causing a dysfunction in the neuronal activation of these fibers [23]. Second, the ketogenic state induced by DM might have contributed to migraine prevention. Ketogenic diet therapy has shown beneficial effects in patients with migraine [24], and the effect of a ketogenic diet on migraine seems to be independent of reduced weight and fat mass [25]. Third, antidiabetic medications may have had a preventive effect on the occurrence of migraine. Lu et al. showed that AMP-activated protein kinase (AMPK) activation reduced neuroinflammation in a mouse model with recurrent nitroglycerin (NTG)-induced chronic migraine (CM), suggesting that metformin, a well-known AMPK activator, could have a preventive effect on migraine occurrence [26]. Similarly, in mice with NTG-induced CM, the glucagon-like peptide-1 (GLP-1) receptor agonist liraglutide attenuated pain hypersensitivity by stimulating interleukin (IL)-10 [27]. Since GLP-1 is also involved in migraine mechanisms, GLP-1 receptor agonists are likely to have potential preventive effects on headache disorders [28]. Other diabetes medications besides GLP-1s may also influence migraine. Metformin, dipeptidyl peptidase-4 (DPP-4) inhibitors, and sodium-glucose transport protein-2 (SGLT-2) inhibitors, which are widely used diabetes treatments, have various effects, including anti-inflammatory properties that impact a range of conditions [29–31]. Neurogenic inflammation is recognized as an important pathophysiological process in migraine, accompanied by various immunological changes [32]. Although the effects of metformin, DPP-4 inhibitors, and SGLT-2 inhibitors on migraine have not been specifically reported, the use of these medications has been associated with a lower risk of depression, a common comorbidity of migraine [33]. In addition to their direct effects, antidiabetic medications may also help reduce obesity, a significant exacerbating factor in migraine. Weight reduction may, in turn, enhance the efficacy of antimigraine medications by reducing their metabolism [5].

Conversely, a positive association was observed between the history of migraine and developing DM in the cohort studies, and the odds of having DM were increased in patients with MO in the cross-sectional studies. The reasons for this inverse relationship are currently unclear. However, the two conditions share many common features. Both conditions involve inflammatory processes and cause elevated proinflammatory markers, such as tissue necrosis factor-α, IL-1β, or IL-6 [32, 34]. Proinflammatory states are known to increase insulin resistance, which increases the incidence of diabetes [35]. The increased risk of DM in individuals with depression, autoimmune conditions, and cardiovascular diseases is explained by the mechanism of a heightened proinflammatory state in patients with these conditions [36–39]. Additionally, some migraine drugs may contribute to inducing DM. A recent randomized clinical trial found that sumatriptan reduced insulin sensitivity and glucose effectiveness in overweight humans [40], and a cohort study found that valproate was associated with a higher risk of developing type 2 diabetes in adults [41].

Genetic composition analysis allows for the investigation of disease associations [42]. Siewert et al. reported a connection between migraine and type 2 diabetes through cross-trait linkage disequilibrium regression, a method used to estimate trait heritability and genetic correlation from genome-wide association study results involving migraine and multiple traits [43]. However, another genetic analysis method, Mendelian randomization (MR), which assesses the causal effect of exposure on outcomes by examining genetic variation, did not find a significant relationship. A study by Xue et al. used MR analysis to examine the associations between Alzheimer’s disease (AD), diabetes, migraine, and multiple sclerosis [44]. They identified a significant link between AD and type 2 diabetes, but not with migraine. Regarding the relationship between migraine and type 2 diabetes, it is anticipated that further genetic composition analyses using various methods in diverse populations will provide deeper insights into the connection between these two conditions.

This study has few limitations. First, the study focused solely on epidemiological studies, including only cross-sectional and cohort studies, and excluded all other research types. This resulted in the inclusion of a reduced number of studies in the meta-analysis, and the possibility of publication bias cannot be ruled out. Second, many studies included in this review relied on diagnostic codes or self-reported diagnoses. While this limitation is somehow inherent to the nature of epidemiological studies, it could compromise the accuracy of diagnoses and is an important factor to consider when interpreting the results. Third, significant heterogeneity was observed in the meta-analysis results, which can be attributed to the varying methodologies employed across the included studies. While we aimed to maintain comparability by including only results that were at least adjusted for age and sex, it is important to note that the extent and type of adjustments for other potential confounders varied among the studies. Additionally, the potential non-linear relationships between DM, migraine, and these confounders may have further influenced the results. Despite our efforts to standardize the analysis, these variations remain a limitation that should be considered when interpreting our findings.

## Conclusions

The study findings showed that DM was negatively associated with the occurrence of migraine, whereas migraine was positively associated with the occurrence of DM. This relationship likely involves various pathophysiological factors as well as medications. This insight can provide new perspectives to physicians who manage these two lifelong conditions and may suggest mechanisms for new treatments for both diseases. However, since most of the results indicated a low or very low level of evidence, they should be interpreted with caution. Future research should focus on conducting long-term follow-ups with more data and on exploring the exact mechanisms underlying these relationships.

## Electronic supplementary material

Below is the link to the electronic supplementary material.

Supplementary Material 1

## Data Availability

No datasets were generated or analysed during the current study.

## Declarations

MKC serves as a board member of *The Journal of Headache and Pain*. MKC was the site investigator for a multicenter trial sponsored by Biohaven Pharmaceuticals, Allergan Korea, and the Ildong Pharmaceutical Company. He has received lecture honoraria from Eli Lilly and Company, Handok-Teva, and the Ildong Pharmaceutical Company over the past 24 months. He received grants from the Yonsei University College of Medicine (6-2021-0229), the Korea Health Industry Development Institute (KHIDI) (HV22C0106), and an NRF grant from the Korean government (MSIT) (2022R1A2C1091767).

## Abbreviations

AD: Alzheimer’s disease
CI: Confidence intervals
DM: Diabetes mellitus
DPN: Diabetic peripheral neuropathy
DPP-4: Dipeptidyl peptidase-4
GLP-1: Glucagon-like peptide-1
GRADE: Grading of Recommendations Assessment Development and Evaluation
HR: Hazard ratios
ICHD: International Classification of Headache Disorders
MA: Migraine with aura
MO: Migraine without aura
MR: Mendelian randomization
NOS: Newcastle–Ottawa Quality Assessment Scale
NTG: Nitroglycerin
OR: Odds ratios
PRISMA: Preferred Reporting Items for Systematic Reviews and Meta-Analyses
SGLT-2: Sodium-glucose transport protein-2
