# Supplementary material for: Epidemiological linkage between migraine and diabetes mellitus: a systematic review and meta-analysis
Source: J Headache Pain. 2024 Sep 27;25(1):158. doi: 10.1186/s10194-024-01868-2 (PMC11438040; doi:10.1186/s10194-024-01868-2)
Supplement: Supplementary file 1 — Supplementary Material 1 [file 10194_2024_1868_MOESM1_ESM.docx]

**ONLINE-ONLY SUPPLEMENTARY MATERIAL**

**Supplementary Material S1.** Search strategy

**Supplementary Material S2.** List of studies excluded during the full-text screening

**Supplementary Material S3.** Adapted form of the Newcastle–Ottawa Quality Assessment Scale for cross-sectional studies

**Supplementary Material S4.** Detailed risk of bias assessment scores using the Newcastle-Ottawa Quality Assessment Scale

**Supplementary Material S5.** Certainty of evidence based on the Grading of Recommendations Assessment, Development and Evaluation (GRADE) framework for each outcome.

**Supplementary Material S6.** Funnel plot analysis for the meta-analysis of the odds of having migraine in individuals with diabetes mellitus

**Supplementary Material S7.** Funnel plot analysis for the meta-analysis of the odds of having diabetes mellitus in individuals with migraine

**Supplemental Material S1.** Search strategy

| **Pubmed 2024-06-02; 505 articles** |
| --- |
| ("diabetes mellitus"[MeSH Terms] OR ("diabetes"[All Fields] AND "mellitus"[All Fields]) OR "diabetes mellitus"[All Fields]) AND ("migrain"[All Fields] OR "migraine disorders"[MeSH Terms] OR ("migraine"[All Fields] AND "disorders"[All Fields]) OR "migraine disorders"[All Fields] OR "migraine"[All Fields] OR "migraines"[All Fields] OR "migraine s"[All Fields] OR "migraineous"[All Fields] OR "migrainers"[All Fields] OR "migrainous"[All Fields]) |
| **Scopus 2024-06-02; 2,996 articles** |
| TITLE-ABS-KEY (diabetes AND mellitus AND migraine) |
| **Web of science 2024-06-02; 343 articles** |
| ALL=(diabetes mellitus migraine) |

**Supplemental Material S2.** List of studies excluded during the full-text screening

**Does not address the topic or relevant outcome, 12**

1. Buse DC, Manack A, Serrano D, Turkel C, Lipton RB. Sociodemographic and comorbidity profiles of chronic migraine and episodic migraine sufferers. J Neurol Neurosurg Psychiatry. 2010 Apr;81(4):428-32. doi: 10.1136/jnnp.2009.192492.
2. Guldiken B, Guldiken S, Taskiran B, Koc G, Turgut N, Kabayel L, Tugrul A. Migraine in metabolic syndrome. Neurologist. 2009 Mar;15(2):55-8. doi: 10.1097/NRL.0b013e31817781b6.
3. Gur-Ozmen S, Karahan-Ozcan R. Factors Associated with Insulin Resistance in Women with Migraine: A Cross-Sectional Study. Pain Med. 2019 Oct 1;20(10):2043-2050. doi: 10.1093/pm/pnz055.
4. Jiménez-García R, López-de-Andrés A, de Miguel-Diez J, Zamorano-León JJ, Carabantes-Alarcón D, Noriega C, Cuadrado-Corrales N, Pérez-Farinos N. Time Trends and Sex Differences in the Association between Diabetes and Chronic Neck Pain, Chronic Low Back Pain, and Migraine. Analysis of Population-Based National Surveys in Spain (2014-2020). J Clin Med. 2022 Nov 25;11(23):6953. doi: 10.3390/jcm11236953.
5. Kurth T, Rist PM, Ridker PM, Kotler G, Bubes V, Buring JE. Association of Migraine With Aura and Other Risk Factors With Incident Cardiovascular Disease in Women. JAMA. 2020 Jun 9;323(22):2281-2289. doi: 10.1001/jama.2020.7172.
6. Lipton RB, Reed ML, Kurth T, Fanning KM, Buse DC. Framingham-Based Cardiovascular Risk Estimates Among People With Episodic Migraine in the US Population: Results from the American Migraine Prevalence and Prevention (AMPP) Study. Headache. 2017 Nov;57(10):1507-1521. doi: 10.1111/head.13179.
7. Liu Y, Gao X, Yuan L, Li Y, Hong P. The Relationship between Triglyceride Glucose Index and Migraine: A Cross-Section Study from the National Health and Nutrition Examination Survey (NHANES). Curr Neurovasc Res. 2023;20(2):230-236. doi: 10.2174/1567202620666230606100652.
8. Magdy R, Othman AS, Elsebaie EH, Elsayed RM, Abdelrahman W, Shalaby S, Saraya M, El-Sayed Abd El-Ghani S, Ayoub YK, Elshall A, Elmazny A. Comorbid conditions in Egyptian patients with migraine. Neurol Res. 2023 Dec;45(12):1100-1110. doi: 10.1080/01616412.2023.2257418.
9. Siva ZO, Uluduz D, Keskin FE, Erenler F, Balcı H, Uygunoğlu U, Saip S, Göksan B, Siva A. Determinants of glucose metabolism and the role of NPY in the progression of insulin resistance in chronic migraine. Cephalalgia. 2018 Oct;38(11):1773-1781. doi: 10.1177/0333102417748928.
10. Tian S, Cheng Z, Zheng H, Zhong X, Yu X, Zhang J, Wu L, Wu W. Interaction between diabetes and body mass index on severe headache or migraine in adults: a cross-sectional study. BMC Geriatr. 2024 Jan 19;24(1):76. doi: 10.1186/s12877-024-04657-3.
11. Wang K, Mao Y, Lu M, Ding Y, Li Z, Li Y, Liu X, Sun Y, Hong J, Xu D, Wu T. Association between migraine and cardiovascular disease: A cross-sectional study. Front Cardiovasc Med. 2022 Nov 24;9:1044465. doi: 10.3389/fcvm.2022.1044465.
12. Wang X, Li X, Diao Y, Meng S, Xing Y, Zhou H, Yang D, Sun J, Chen H, Zhao Y. Are Glucose and Insulin Metabolism and Diabetes Associated with Migraine? A Community-Based, Case-Control Study. J Oral Facial Pain Headache. 2017 Summer;31(3):240-250. doi: 10.11607/ofph.1843

**Does not adjust for age or sex, 1**

1. Rieder A, Lobentanz I, Zeitlhofer J, Mitsche N, Lawrence K, Schwarz B, Kunze M. Background morbidity of headache in an adult general population. Results of the Austrian SERMO (Self-Reported Morbidity) study. Wien Klin Wochenschr. 2004 Mar 31;116(5-6):176-81. doi: 10.1007/BF03040484.

**Case-control study, 5**

1. Burn WK, Machin D, Waters WE. Prevalence of migraine in patients with diabetes. Br Med J (Clin Res Ed). 1984 Dec 8;289(6458):1579-80. doi: 10.1136/bmj.289.6458.1579-a.
2. Couch JR, Hassanein RS. Headache as a risk factor in atherosclerosis-related diseases. Headache. 1989 Jan;29(1):49-54. doi: 10.1111/j.1526-4610.1989.hed2901049.x.
3. Gilad R, Boaz M, Dabby R, Finkelstein V, Rapoport A, Lampl Y. Migraine and vascular risk factors in the elderly. Geriatr Gerontol Int. 2014 Jan;14(1):220-5. doi: 10.1111/ggi.12061.
4. Haghighi FS, Rahmanian M, Namiranian N, Arzaghi SM, Dehghan F, Chavoshzade F, Sepehri F. Migraine and type 2 diabetes; is there any association? J Diabetes Metab Disord. 2016 Sep 8;15(1):37. doi: 10.1186/s40200-016-0241-y.
5. Özcan RK, Özmen SG. The Association Between Migraine, Metabolic Syndrome, Insulin Resistance, and Obesity in Women: A Case-Control Study. Sisli Etfal Hastan Tip Bul. 2019 Nov 20;53(4):395-402. doi: 10.14744/SEMB.2018.09582.

**Case series, 1**

1. Blau JN, Pyke DA. Effect of diabetes on migraine. Lancet. 1970 Aug 1;2(7666):241-3. doi: 10.1016/s0140-6736(70)92588-2.

**Supplemental Material S3.** Adapted form of the Newcastle–Ottawa Quality Assessment Scale for cross-sectional studies

**Selection: (Maximum 4 scores)**

1) Representativeness of the cases:

a) Truly representative of the average patients with migraine/diabetes in the community (random sampling of cases). 1 score

b) Somewhat representative of the average patients with migraine/diabetes in the community (non-random sampling). 1 score

c) Selected demographic group of users. 0 score

d) No description of the sampling strategy. 0 score

2) Sample size:

a) Justified and satisfactory. 1 score

b) Not justified. 0 score

c) No information provided. 0 score

3) Non-respondents

a) The response rate is satisfactory, and the comparability between respondents and non-respondents characteristics is established. 1 score

b) The response rate is unsatisfactory, or the comparability between respondents and non-respondents is not established, or no description. 0 score

4) Ascertainment of the exposure:

a) Validated screening/surveillance tool or structured review. 1 score

b) Written self-report. 0 score

c) No description. 0 score

d) Other. 0 score

**Comparability: (Maximum 2 scores)**

1) The potential confounders were investigated by subgroup analysis or multivariable analysis.

a) The study controls for age and sex. 1 score

b) Study controls for other factors, such as body mass index, race/ethnicity, income, educational level or exercise habits. 1 score

**Outcome: (Maximum 3 scores)**

1) Assessment of the outcome:

a) Independent blind assessment. 2 scores

b) Record linkage. 2 scores

c) Self report. 1 score

d) No description. 0 score

2) Statistical test:

a) The statistical test used for data analysis was clearly described and appropriate; the measurement of the association was also presented. 1 score

b) The statistical test is not appropriate, or not described. 0 score

**Supplemental Material S4.** Detailed risk of bias assessment scores using the Newcastle-Ottawa Quality Assessment Scale

**Cross-sectional studies**

| **1st Author** | **Year** | **Selection** | | | | **Comparability** | **Outcome** | | **Final score** |
| --- | --- | --- | --- | --- | --- | --- | --- | --- | --- |
|  |  | 1) Representativeness of the cases | 2) Sample size | 3) Non-respondents | 4) Ascertainment of the exposure |  | 1) Assessment of outcome | 2) Statistical test |  |
| Aamodt | 2007 | + | - | + | + | + | + | + | 6 |
| Berge | 2013 | + | - | + | + | + | - | + | 7 |
| Bigal | 2010 | + | - | + | + | + | + | + | 6 |
| Hagen | 2018 | + | - | + | + | + | + | + | 6 |
| López-de-Andrés | 2018 | + | - | - | + | + | + | + | 5 |
| Minen | 2019 | + | - | - | + | + | + | + | 5 |
| Patel | 2019 | - | - | + | + | + | - | + | 6 |
| Schramm | 2021 | + | - | - | + | + | - | + | 6 |

**Cohort studies**

| **1st Author** | **Year** | **Selection** | | | | **Comparability** | **Outcome** | | | **Final score** |
| --- | --- | --- | --- | --- | --- | --- | --- | --- | --- | --- |
|  |  | 1) Representativeness of the exposed cohort | 2) Selection of the non-exposed cohort | 3) Ascertainment of exposure | 4) Demonstration that outcome of interest was not present at start of study |  | 1) Assessment of outcome | 2) Was follow-up long enough for outcomes to occur | 3) Adequacy of follow up of cohorts |  |
| Antonazzo | 2018 | + | + | + | + | + | + | + | + | 8 |
| Burch | 2012 | - | + | + | + | + | + | + | - | 6 |
| Fagherazzi | 2019 | - | + | + | + | + | + | + | + | 7 |
| Wu | 2024 | - | + | + | + | + | + | + | - | 6 |

**Supplemental Material S5.** Certainty of evidence based on the Grading of Recommendations Assessment, Development and Evaluation (GRADE) framework for each outcome.

| **Study type** | **Outcomes** | **No of studies** | **Results (95% CI)** | **Quality assessment** | | | | | **Certainty of Evidence** |
| --- | --- | --- | --- | --- | --- | --- | --- | --- | --- |
|  |  |  |  | Study design* | Inconsistency† | Indirectness‡ | Imprecision§ | Other consideration |  |
| Cross-sectional | DM to migraine | 4 | OR 0.85 (0.69, 1.05) | not serious | serious | not serious | serious | none | **VERY LOW** |
|  | Type 1 diabetes to migraine | 2 | OR 0.48 (0.30, 0.77) | not serious | not serious | not serious | not serious | none | **LOW** |
|  | Type 2 diabetes to migraine | 2 | OR 0.87 (0.59, 1.26) | not serious | serious | not serious | serious | none | **VERY LOW** |
|  | Migraine to DM | 4 | OR 1.00 (0.73, 1.37) | not serious | serious | not serious | serious | none | **VERY LOW** |
|  | MA to DM | 2 | OR 0.91 (0.33, 2.47) | not serious | serious | not serious | serious | none | **VERY LOW** |
|  | MO to DM | 2 | OR 1.19 (1.02, 1.39) | not serious | not serious | not serious | serious | none | **VERY LOW** |
| Cohort | DM to migraine | 2 | HR 0.83 (0.76, 0.90) | not serious | not serious | not serious | not serious | none | **LOW** |
|  | Type 1 diabetes to migraine | 1 | HR 0.74 (0.61, 0.89) | not serious | not serious | not serious | not serious | none | **LOW** |
|  | Type 2 diabetes to migraine | 2 | HR 0.85 (0.80, 0.91) | not serious | not serious | not serious | not serious | none | **LOW** |
|  | Migraine to DM | 2 | HR 0.98 (0.85, 1.14) | not serious | serious | not serious | serious | none | **VERY LOW** |
|  | Active migraine to DM | 2 | HR 0.86 (0.59, 1.25) | not serious | not serious | not serious | serious | none | **VERY LOW** |
|  | Past migraine to DM | 2 | HR 1.09 (1.01, 1.17) | not serious | not serious | not serious | serious | none | **VERY LOW** |

DM, diabetes mellitus; OR, odds ratio; HR, hazards ratio; CI, confidence interval; MO, migraine without aura; MA, migraine with aura

* Downgraded by one level if >25% of participants in this comparison were from studies at high risk of bias.

† Downgraded by one level if heterogeneity (I^2^) >50%.

‡ Downgraded by one level if I^2^ >25% of included studies were center-based.

§ Downgraded by one level if the 95% CI for risk estimates cross a minimally important difference of 5% for outcomes (OR or HR 0.95–1.05).


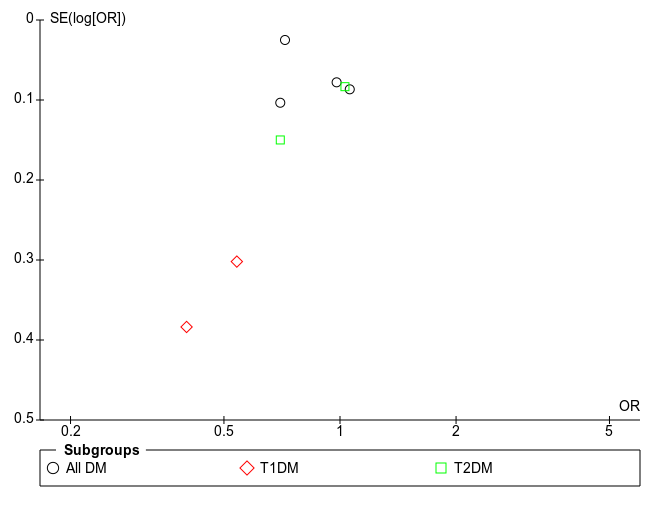


**Supplemental Material S6.** Funnel plot analysis for the meta-analysis of the odds of having migraine in individuals with diabetes mellitus


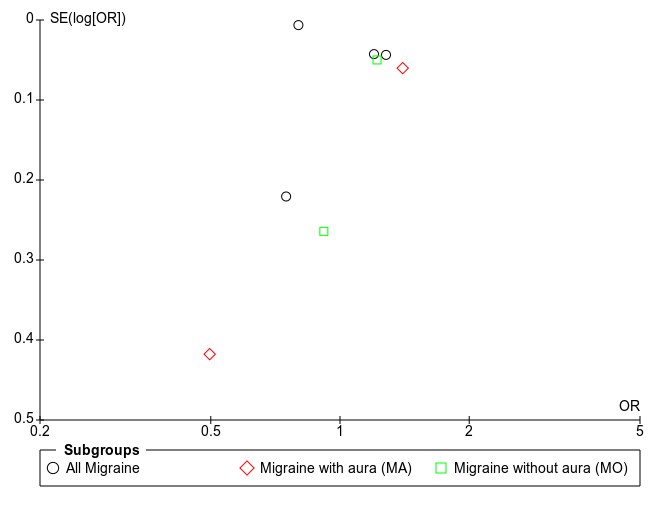


**Supplemental Material S7.** Funnel plot analysis for the meta-analysis of the odds of having diabetes mellitus in individuals with migraine
